# Nonfatal Occupational Injuries among Workers in Microscale and Small-Scale Woodworking Enterprise in Addis Ababa, Ethiopia

**DOI:** 10.1155/2020/6407236

**Published:** 2020-01-31

**Authors:** Hailemichael Mulugeta, Yifokire Tefera, Meaza Gezu

**Affiliations:** ^1^College of Health Science, Debre Berhan University, Debre Berhan, Ethiopia; ^2^School of Public Health, College of Health Sciences, Addis Ababa University, Addis Ababa, Ethiopia

## Abstract

**Background:**

Microscale and small-scale industries have been widely expanded in low-income countries, including Ethiopia, as a job opportunity for young workers, which makes workers vulnerable to injury. Woodworking is one of the high-risk jobs in this sector due to the use of hazardous tools and machineries. Therefore, the aim of this study was to estimate the prevalence of injury and associated contributing factors of this sector.

**Methods:**

A cross-sectional study design was conducted among 634 workers selected from 194 microscale and small-scale woodworking enterprises in Addis Ababa. Data were collected using a structured interview questionnaire and observation checklist from February to March 2016. Occupational injuries were documented according to the ILO operational definition. Descriptive statistics and multivariable analyses were used to characterize the data and to identify the factors associated with injury at a *p* value <0.05, respectively.

**Result:**

A total of 625 (98.6%) workers were interviewed. The prevalence of occupational injury was 92 (14.7%) in the past 12 months. Workers with khat chewing behavior (AOR: 2.25, 95% CI (1.04, 4.85)), job dissatisfaction (AOR: 2.89, 95% CI (1.75, 4.76)), work-related stress (AOR: 4.79, 95% CI (1.69–13.58)), job categories (AOR: 3.52, 95% CI (1.08, 11.41)) and workplace characteristics such as unguarded machines (AOR: 3.32 (1.21, 9.11)), and inadequate work space (AOR: 3.85 (1.14, 13.04)) were significantly associated with occupational injury.

**Conclusion:**

The prevalence of nonfatal occupational injuries among workers in this study was substantially high. Workers' behavior, psychosocial issues, and work-related characteristics played a causal role in the occurrence of occupational injury. Therefore, workers' safety protection and behavioral intervention should be initiated.

## 1. Introduction

Microscale and small-scale enterprises (MSEs) are expanding strategically in low-income countries as a means of job opportunity for young workers [1]. These enterprises are expected to increase from 14.6% in 2015 to 22.8% by the end of 2020 in Ethiopia [2]. The Ethiopian government has given special attention to the expansion of MSEs due to the additional benefits for industrialization [2–5]. According to the Ethiopian MSE Development Strategy, individuals not completing formal education, vocational training graduates, and less-skilled citizens are advised to form cooperatives of MSE with the goal of creating about 4 million new jobs in the Ethiopian Growth and Transformation period [2, 4]. The MSEs are classified into microscale and small-scale enterprises based on their number of employees, being less than 6 and 6–30, respectively [6]. Micro and small woodworking enterprises are categorized under the manufacturing sector. Though the MSE are considered as an informal economy, these business units employ more than 5.5 million people with the majority being youth [5–8].

The machineries used and the working conditions in the woodworking activities are very dangerous and may lead to serious occupational injuries [9]. The presence of rotating devices, unguarded cutting, or shearing blades is the sources of injury in wood workshops [10]. Since the sector is considered as an informal economy, the rights of workers, labor inspection services, access to occupational health services, and injury recording or notification were significantly compromised [5]. The prevention of occupational injury needs appropriate monitoring, regulation, capacity, and commitment of workplace administrators [11, 12].

Several studies from low-income settings reported higher prevalence of occupational injuries in the MSE manufacturing sector. Occupational injury prevalence studies in Ethiopia [13–15], Ghana [12, 16], and Zimbabwe [10] revealed that workers are highly vulnerable to injury in this sector. Studies also showed that work-related conditions [13, 14, 16] such as contact with machinery [16], work-related stress [10], job dissatisfaction [14], and inadequate work space [17] were significantly associated with occupational injury. In addition, workers' behavioral characteristics such as chewing khat [14], smoking [14, 16] and lack of personal protective equipment use [14, 15] were also showed significant association with injuries.

Most of the above studies were conducted in the general MSE manufacturing sectors but little is known specifically in the woodworking sector. Hence, evidence for planning intervention activities in the micro and small woodworking has been historically limited. Therefore, the aim of this study was to estimate the prevalence of occupational injuries and the associated factors among workers in the microscale and small-scale woodworking enterprises.

## 2. Materials and Methods

Institution-based cross-sectional study design was used which lasted from February 17 to March 14, 2016, among workers of microscale and small-scale woodworking enterprises in Addis Ababa. It is the capital city of Ethiopia, which has 10 subcities (boroughs). Addis Ababa was selected due to the presence of a large number of woodworking enterprises and the engagement of work force compared to other towns in Ethiopia [8, 18]. A total of 3,339 and 613 microscale and small-scale woodworking enterprises were found in Ethiopia and Addis Ababa, respectively [19].

There is no previous study in Ethiopia on the prevalence of nonfatal occupational injuries among workers in microscale and small-scale woodworking enterprises. Hence, prevalence of injury of 50% was used to estimate the sample. A sample size of 634 workers was calculated using a single-population proportion formula with the assumption of 50% prevalence, 5% margin of error, 95% confidence level, 1.5 design effect, and 10% added for nonresponse.

The sampling procedure was done through two steps. First five subcities out of the 10 subcities were selected using a lottery method. Then, the sample size was allocated to microscale and small-scale woodworking enterprises using proportional allocations to the size to determine the numbers of study subjects required from each selected subcity. A sampling frame (list) of enterprises was taken from the Addis Ababa City Administration Bureau of Trade's central database [19]. Subsequently, the enterprises were selected randomly with Excel software until the required sample size was obtained. A total of 634 workers who were directly engaged in the woodworking production process (423 from micro and 211 from small enterprises) were selected to participate in the study. Workers who were absent from work for any reason during the time of data collection were excluded from the study.

A pretested and structured questionnaire was used to collect information from workers, and an observation checklist was used to collect the work environment data. The interview questionnaire and observation checklist were developed based on published literature and previous studies of similar setting [13, 20–25]. The questionnaire included assessment tools of the Marlin Company and the American Institute of Stress Scale [22], the Generic Job Satisfaction Scale [23], and ILO operational definition for occupational injury [21]. Both the Stress Scale and Job Satisfaction Scale were validated in previous studies and translated into Amharic, the local language [20]. In the present study, nonfatal occupational injuries were an outcome variable. The injury types were classified based on the International Statistical Classification of Diseases and Related Health Problems [26]. Workers were interviewed about their sociodemographic, behavioral, and work-related characteristics: sex, age, work experience, salary, education (illiterate, read and write, primary, secondary, college, and above), sleep hours per day, khat chewing (yes/no), cigarette smoking (yes/no), alcohol drinking (yes/no), psychosocial factors (work-related stress and job satisfaction), type of employment (permanent/temporary), job category (painter, machine operator and carpenter), work hour per week, night work (yes/no), use of personal protective equipment (yes/no), and training on health and safety (yes/no). In addition, the observation checklist was used to examine the adequacy of working space (yes/no), the presence of an obstacle on the floor (yes/no), the adequacy of lighting (yes/no), the presence of machine guarding (yes/no), the presence of reachable emergency power cutoff switch (yes/no), the presence of functional fire extinguisher (yes/no), and availability of personal protective equipment (PPE) (yes/no).

One public health practitioner served as a supervisor, and three occupational health and safety professionals participated as data collectors. Two days of training were given for the data collectors concerning the study objective, tools, approach, and ethical issues. A pretest was done among 5% of the sample size in other subcities to check the consistency. Interviews were conducted in a private space or room to maintain the privacy of the respondent and to avoid disturbance. Observation data were collected with a checklist on the same day after interviewing the workers. Data collection activities were carried out with close follow-up by the principal investigators and the supervisor.

The data were entered into an electronic template of Epinfo version 7.1 and analyzed using SPSS version 20. The data from the interview and observation were analyzed independently. There were many independent variables from the interview; hence, variables with a *p* value below 0.3 with the outcome variable in the bivariate analysis were entered into the multivariable logistic regression model [20, 27, 28]. The required assumptions of the logistic regression were checked with Hosmer and Lemeshow fit test statistics. Odds ratio (OR) with 95% CI was used to declare the presence and the strength of association in the multivariable analysis.

The study protocol was reviewed and approved by the Institutional Review Board of Addis Ababa University Research and Ethical Committee of the School of Public Health. Permission was granted by each enterprise owner before approaching workers. Informed written consent was obtained from each participant for the study and dissemination of the results. Participation in this study was voluntary. Confidentiality was ensured for the collected information through the use of coding.

### 2.1. Operational Definitions and Measurement

  Self-reported occupational injuries include injuries that occurred due to incidents at the workplace during the past 12 months; and those injured workers should have been away from work at least one day because of the injury, in addition to the day of the incident [21, 24].
  Alcohol drinking is consumption of any alcohol more than twice a week [13].
  Khat (pronounced “cot”) is a stimulant drug derived from a shrub (*Catha edulis*) that is native to East Africa and southern Arabia. Leaves of the khat shrub are typically chewed and held in the cheek, like chewing tobacco, to release their stimulant chemicals [29].
  Khat chewing refers to chewing khat leaves before or while working more than twice a week.
  Smoking is referred to smoking at least one stick of tobacco cigarette each day.
  Workplace stress status is a result with a total score of 26 or more for work stress presence and less than 26 for no work stress [22].
  Job satisfaction status is a result with total score 32–50 as satisfied with the job and less than 32 as no job satisfaction [23].
  Adequate work space is the working area with enough clear space (walkways and around cupboards, storage, or doors) to allow physical actions needed to perform the task [30].
  Obstacle-free floor include floors maintained to be free of slip and trip hazards (trailing cables, uneven edges or broken surfaces) and walkways clear and free from tools and materials [30].
  Adequate light includes lighting is sufficient to allow workers to see machinery movements, controls, displays, and to move about easily and free of flicker and glare [30, 31].
  Guard is a part of machinery specifically designed to provide protection by means of a physical barrier [32].
  Personal protective equipment (PPE) includes goggles, helmet, and face shield and gloves, boots, and specialized clothing that is designed to protect parts of the body including the eyes, face, hands, figure, and feet [9].

## 3. Results

### 3.1. Sociodemographic Characteristics of Respondents

A total of 625 respondents were interviewed from 162 microscale and 32 small-scale woodworking enterprises (416 and 209 workers from microscale and small-scale workers, respectively) with a response rate of 98.6%. The majority of the participants were male (94.6%), age less than 30 (75.5%), and with a work experience of less than 5 years (64.2%) (Table 1).

### 3.2. Behavioral and Psychosocial Characteristics of Respondents

Of the total respondents, 8.6% drank alcohol, 8.8% chewed khat, and 4.3% smoked cigarettes. The overall use of PPE was low (20.8%). Out of the total PPE users, the majority (82.4%) used only goggles. The reasons for not using were identified as lack of availability (75.8%), discomfort to use (21.2%), and the use of PPE limiting work performance (3.0%). Regarding psychosocial characteristics, 25.9% and 3% of the participants reported being dissatisfied with their job and had work-related stress, respectively.

### 3.3. Work-Related Characteristics of Respondents

About half of the participants were working with a contractual agreement. The majority (93.9%) work during daytime, 23.8% work >48 hour per week, about half (55.8%) of the workers had training on health and safety, and 39.4% worked as both machine operator and carpenter.

### 3.4. Work Environment Characteristics

A total of 162 (54.9%) microscale and 32 (59.2%) small-scale woodworking enterprises were observed. The majority of the enterprises 158 (81.4%) had unguarded machinery, and 154 (79.4%) did not provide adequate working space (Table 2).

### 3.5. Prevalence of Nonfatal Occupational Injuries

The prevalence of nonfatal occupational injury among the 625 participants was 14.7% during the past 12 months. This prevalence among microscale and small-scale enterprises was similar (14.9% and 14.4%, respectively). About half (52%) of the injuries resulted in the workers being absent from work for more than 3 days. Cut or laceration were the most prevalent type of injury (63%), and fingers were the most affected body parts (68.5%).

Most injures happened due to contact to exposed machinery (68.5%) while cutting, flattening, and smoothing timbers. The highest number of injuries was reported on Mondays (33.7%) and in the morning (51.1%) (Table 3).

### 3.6. Factors Associated with Nonfatal Occupational Injury

Each variable was analyzed using bivariate logistic regression, and variables with a *p* value of less than 0.3 were fitted to the multivariable logistic regression. Variables including work experience, monthly salary, educational status, sleeping hours per day, employment pattern, working hour per week, night work, health and safety training, and type of enterprise were not fitted (*p* < 0.3). In the multivariable analysis, individual characteristics such as khat chewing, job satisfaction, and work-related stress and job category were significantly associated with the occurrence of nonfatal occupational injury at *p* < 0.05 (Table 4).

Similarly, variables of the work environment characteristics such as unguarded machines and inadequate work space were significantly associated with the occurrence of occupational injury (Table 5).

## 4. Discussion

The prevalence of nonfatal occupational injuries in this study was 14.7% (147 per 1000 workers). Previous studies in Ethiopia had reported higher injury prevalence ranging from 452 to 808 per 1000 exposed workers per year in the microscale and small-scale enterprises [13–15]. It could be difficult to compare our study prevalence estimate with the above studies due to the use of different case definitions to count an injury. The use of a definition “at least one day absence from work after an injury” in this study might lower the prevalence in the current study. An injury definition in the workplace can significantly affect the prevalence estimate [33]. To avoid overreporting of minor injuries like scratches and abrasions, most countries used a reportable injury definition as “which can leave the victim absent from work for three and more days” [34]. However, such definition might be difficult to use in an informal sector where the majority of the workers were temporary employees and had no insurance access.

This study indicated that the upper limbs, including hands and fingers were the most commonly affected parts of the body. Several studies also reported similar findings among the manufacturing workers [13, 14, 35, 36]. Most of the tasks in woodworking were performed by the use of machinery and hand tools. Hence, hands are frequently in contact with sharp tools which increase the risk of injury. The majority of the injuries reported in the current study were one-time occurrences. It is similar to other occupational injury studies [20, 36, 37]. This could be due to workers who might learn a preventive behavior after experiencing injury [38] or after major injury, workers may be afraid to go back to work again. That is why, the ILO recommended injury recording, notification, and injury surveillance system at the workplace [39].

Contact with machinery was found to be the main cause of injury in this study. A significant percentage of the machines had unguarded parts, and workers used defective hand tools in the current study. Similar results have been reported in other studies at small- and medium-scale industries [36, 37]. Injuries due to table saw use were found to be the most common as compared to other types of machineries among injured participants. This could be because of the frequent use of these machines in the woodworking enterprises. Hence, the probability of getting injured might be increased while operating these machines without appropriate safety measures [40, 41]. The majority of MSE had inadequate working space and no guards on hazardous parts of machinery which showed significant association with injury inccident in the study. This finding is consistent with a study in Malaysia [17]. Inadequate work space could limit worker movement and increase involuntary contact of workers with unguarded machines or hazardous objects. The injuries could occur while the workers became close to the rotating motion of tools [42].

The current study revealed that individual and behavioral characteristics are associated with occupational injury. The probability of injury occurrence among khat chewers, machine operators, and carpenters was significantly high. Other studies in Ethiopia have showed an increased risk of injury among khat chewers [14, 43]. Khat is classified as addictive substance which stimulates and increases excitement that later can affect the central nervous system [29, 44]. Studies of cognitive tests demonstrated that khat chewers perform worse as compared to nonusers [45] and the levels of anxiety and depression were higher among chewers than nonchewers [46]. These health effects might impair worker concentration and increase the likelihood of mistakes and occurrence of accidents at work.

Psychosocial characteristics such as work-related stress and job dissatisfaction of workers were significantly associated with injury in the present study. Workers who were not satisfied with their job had an injury risk nearly three times higher than workers who reported to be satisfied with their job. This finding was similar to a study conducted on small-scale and medium-scale industries [14, 37, 47]. Unsatisfied individuals might increase the likelihood of making mistakes while working in their job which leads to an injury [48]. Similarly, workers who experienced stress in the work place had more than four times risk to injury. A similar finding was reported among textile factory workers in Ethiopia [20]. The nature of woodworking, poor working condition, the lack of appropriate safety equipment, and lack of safety training for protection might be stressful for workers [22, 49]. Prior research studies indicated that such stressful condition could alter either the mental or physical nature of workers and impair workers focus which can lead to commit error [49, 50]. In addition, the safety behavior of workers could be affected by emotional stress [51].

The use of standard case definition for an injury, high participation rate, the use of standard measurement tools, and involvement of many enterprises were the strengths of this study. However, the findings were based up on self-reporting and injuries were not clinically confirmed. Since MSEs were informal sectors where temporary employment is a common practice, a healthy worker may be affected and severely injured workers may not return to their job.

Generally, the prevalence of nonfatal occupational injuries among workers in MSE woodworking enterprises is substantially high. Contact with unguarded machineries and poor working condition were the major causes of injury incident. Behavioral characteristics of khat chewing, psychosocial characteristics, and job category were significantly associated with occupational injury. Therefore, workplace safety improvement and behavioral change communication activities should be included in the microscale and small-scale woodworking enterprise development strategy.

## Figures and Tables

**Table 1:** Sociodemographic characteristics of respondents in microscale and small-scale woodworking enterprises, Addis Ababa, Ethiopia.

Variable	*N* (%)
Sex	
Male	591 (94.6)
Female	34 (5.4)

Age group	
<30 years	472 (75.5)
≥30 years	153 (24.5)

Educational status	
Illiterate	23 (3.7)
Read and write	17 (2.7)
Primary school [1–8]	236 (37.8)
Secondary school [9–12]	249 (39.8)
College and above	100 (16.0)

Work experience	
<5 year	401 (64.2)
≥5 year	224 (35.8)

Salary per month in Ethiopian Birr (US$)	
<1400 (66.7)	113 (18.1)
1401–2350 (66.7–107.8)	228 (36.5)
2351–3550 (107.8–170.7)	204 (32.6)
3550+ (170.7+)	80 (12.8)

**Table 2:** Occupational safety practice among woodworking enterprise, Addis Ababa, Ethiopia.

Variables	Total (*N* = 194)
	Number (%)
Workplace	
Indoor	114 (58.8)
Indoor and outdoor	80 (3.1)

Adequate work space	
Yes	40 (20.6)
No	154 (79.4)

Obstacle-free floor	
Yes	65 (33.5)
No	129 (66.5)

Adequate light	
Yes	176 (90.7)
No	18 (9.3)

Guarded machine	
Yes	36 (18.6)
No	158 (81.4)

Reachable power cutoff switch	
Yes	186 (95.9)
No	8 (4.1)

Presence of fire extinguisher	
Yes	10 (5.2)
No	184 (94.8)

Availability of PPE for all workers	
Yes	62 (32.0)
No	132 (68.0)

**Table 3:** Characteristics of nonfatal occupational injuries among injured respondents in the woodworking enterprises, Addis Ababa, Ethiopia.

Variables	Number (%)
Nonfatal occupational injury (*n* = 625)	
Yes	92 (14.7)
No	533 (85.3)

Injury frequency (*n* = 92)	
One time	89 (96.8)
Two and more	3 (3.2)

Parts of the body affected (*n* = 92)	
Hand	4 (4.3)
Foot	1 (1.1)
Hand finger	63 (68.5)
Head	3 (3.3)
Lower arm	12 (13.0)
Upper leg	1 (1.1)
Lower leg	8 (8.7)

Cause of injury (*n* = 92)	
Slip	2 (2.2)
Fall-same level	2 (2.2)
Falling object from a height	4 (4.3)
Contact with machine blade	63 (68.5)
Splintering objects	10 (10.9)
Nonpowdered saw	3 (3.2)
Other	8 (8.7)

Activity while injured (*n* = 92)	
Cutting	52 (56.5)
Smoothing, creating patterns, and shaping	22 (23.9)
Other work in production process	18 (19.6)

Day of injuries (*n* = 92)	
Monday	31 (33.7)
Tuesday	7 (7.6)
Wednesday	7 (7.6)
Thursday	9 (9.8)
Friday	3 (3.3)
Saturday	25 (27.2)
Sunday	3 (3.2)
Did not remember	7 (7.6)

Time of injury (*n* = 92)	
Morning (7:00 am–1:30 pm)	50 (54.4)
Afternoon (1:30 pm–5:30 pm)	28 (30.4)
Evening (5:30 pm–10:00 pm)	13 (14.1)
Did not remember	1 (1.1)

Days lost due to injury (*n* = 92)	
≤3 days	40 (43.5)
>3 days	52 (56.5)

**Table 4:** Multivariate analysis of individual factors associated with nonfatal occupational injury in the woodworking enterprises, Addis Ababa, Ethiopia.

Variable name	Injury status (*n* = 625)		Crude OR (95% CI)	Adjusted OR (95% CI)
	Yes (%)	No (%)		
Sex				
Male	90 (15.2)	501 (84.8)	2.87 (0.68–12.2)	1.61 (0.36–7.16)
Female	2 (6.3)	32 (94.1)	1	1

Age				
<30	74 (15.7)	398 (84.3)	1.39 (0.80–2.41)	1.47 (0.80–2.71)
≥30	18 (11.8)	135 (88.2)	1	1

Khat chewing status				
Yes	17 (30.9)	38 (69.1)	2.95 (1.59–5.50)^*∗*^	2.25 (1.04–4.85)^*∗∗*^
No	75 (13.2)	495 (86.8)	1	1

Alcohol drinking status				
Yes	14 (25.9)	40 (74.1)	2.21 (1.15–4.25)^*∗*^	1.35 (0.60–3.05)
No	78 (13.7)	493 (86.3)	1	1

Cigarette smoking status				
Yes	9 (33.3)	18 (66.7)	3.10 (1.35–7.14)^*∗*^	2.20 (0.76–6.37)
No	83 (13.9)	515 (86.1)	1	1

Work-related stress score				
>25	8 (42.1)	11 (57.9)	4.5 (1.77–1.56)^*∗*^	4.79 (1.69–13.58)^*∗∗*^
≤25	84 (13.9)	522 (86.1)	1	1

Job satisfaction scores				
≤31	43 (26.5)	119 (73.5)	3.05 (1.92–4.82)^*∗*^	2.89 (1.75–4.76)^*∗∗*^
>31	49 (10.6)	414 (89.4)	1	1

Any PPE use				
Yes	13 (10.0)	117 (90.0)	0.59 (0.31–1.09)	0.83 (0.43–1.61)
No	79 (16.0)	416 (84.0)	1	1

Night time work				
Yes	9 (23.7)	29 (76.3)	1.88 (0.86–4.12)	1.47 (0.62–3.49)
No	83 (14.1)	504 (85.9)	1	1

Job category				
Painter	4 (5.7)	66 (94.3)	1	1
Machine operator	19 (19.2)	80 (80.8)	1.32 (0.72–2.4)	1.27 (0.66–2.41)
Machine operator and carpentry	38 (15.4)	208 (84.6)	3.97 (1.29–12.24)^*∗*^	3.52 (1.08–11.41)^*∗∗*^
Carpentry	16 (20.0)	64 (80.0)	1.00 (0.46–2.02)	0.97 (0.44–2.15)
All types of work	15 (11.5)	115 (88.5)	1.84 (0.88–3.84)	2.05 (0.93–4.52)

^*∗*^ *p* value <0.05 in bivariate analysis; ^*∗∗*^*p* value <0.05 in multivariate analysis.

**Table 5:** Injuries and associated factors in microscale and small-scale woodworking enterprises, Addis Ababa, Ethiopia.

Variable	Injury incident		COR (95% CI)	AOR (95% CI)
	Yes	No		
Type of enterprises				
Micro MWWE^a^	58 (36%)	104 (64%)	0.82 (0.38–1.77)	1.02 (0.39–2.68)
Small SWWE^b^	13 (41%)	19 (59%)	1	1

Machine-based work place				
Indoor	40 (41%)	57 (59%)	1	1
Indoor and outdoor	31 (32%)	66 (68%)	0.67 (0.37–1.21)	0.62 (0.30–1.27)

Adequate work area				
Yes	6 (15%)	34 (85%)	1	1
No	65 (42%)	89 (58%)	0.00 (1.64–10.43)^*∗*^	3.85 (1.14–13.04)^*∗∗*^

Obstacle-free floor				
Yes	16 (25%)	49 (75%)	1	
No	55 (43%)	74 (57%)	2.23 (1.17–4.41)^*∗*^	0.95 (0.38–2.39)

Adequate light				
Yes	67 (38%)	109 (62%)	1	1
No	4 (22%)	14 (78%)	0.46 (0.15–1.47)	0.90 (0.24–3.40)

Machine guard				
Yes	6 (17%)	30 (83%)	1	1
No	65 (41%)	93 (59%)	3.45 (1.38–8.88)^*∗*^	3.32 (1.21–9.11)^*∗∗*^

Reachable power cutoff switch				
Yes	67 (36%)	118 (64%)	1	1
No	4 (44%)	5 (56%)	1.41 (0.37–5.42)	1.47 (0.33–6.59)

Functional fire extinguisher				
Yes	2 (20%)	8 (80%)	1	1
No	69 (38%)	115 (62%)	2.4 (0.50–11.6)	2.21 (0.41–12.16)

PPE available for workers				
Yes	19 (31%)	43 (69%)	1	1
No	52 (39%)	80 (61%)	1.47 (0.77–2.80)	1.7 (0.84–3.45)

^a^Microscale woodworking enterprises; ^b^Small-scale woodworking enterprises. ^*∗*^*p* value <0.05 in bivariate analysis; ^*∗∗*^*p* value <0.05 in multivariate analysis.

## Data Availability

Data and other required materials can be submitted if requested.
